# Communication Efficient Algorithms for Bounding and Approximating the Empirical Entropy in Distributed Systems

**DOI:** 10.3390/e24111611

**Published:** 2022-11-05

**Authors:** Amit Shahar, Yuval Alfassi, Daniel Keren

**Affiliations:** Department of Computer Science, University of Haifa, Haifa 3498838, Israel

**Keywords:** entropy, entropy approximation, entropy bounds, distributed systems, sketches

## Abstract

The empirical entropy is a key statistical measure of data frequency vectors, enabling one to estimate how diverse the data are. From the computational point of view, it is important to quickly compute, approximate, or bound the entropy. In a distributed system, the representative (“global”) frequency vector is the average of the “local” frequency vectors, each residing in a distinct node. Typically, the trivial solution of aggregating the local vectors and computing their average incurs a huge communication overhead. Hence, the challenge is to approximate, or bound, the entropy of the global vector, while reducing communication overhead. In this paper, we develop algorithms which achieve this goal.

## 1. Introduction

Consider the distributed computing model [1,2,3], where the goal is to compute a function over input divided amongst multiple nodes. A local computation, while simple, does not always suffice to reach a conclusion on the aggregated input data, especially when the function is nonlinear. On the other hand, broadcasting the local data to a coordinator node is impractical and undesirable due to communication overhead, energy consumption, and privacy issues. Generally, we seek to approximate or bound the function’s value on the aggregated data without broadcasting it in its entirety.

The function we handle in this paper is the empirical Shannon entropy [4], which is defined as $\sum_{i}-x_{i}\ln\left(x_{i}\right)$ for a frequency vector $X=\left(x_{1},\ldots ,x_{n}\right)$ (i.e., all values are non-negative and sum to 1). For some of the ensuing analysis, it is easier to use the natural logarithm than the base 2 one, which only changes the value by a multiplicative constant. Thus, hereafter, “entropy” will refer exclusively to the empirical Shannon entropy. Specifically, we assume there exists a distributed system, with each node (or “party”) holding a “local” frequency vector. The target function is defined as the global system entropy, which is equal to the empirical Shannon entropy of the average of the local vectors. Alas, to compute the exact value, we must first aggregate the local vectors and average them, which often incurs a huge communication overhead. Fortunately, it often suffices to approximate, or bound, this global entropy; for example:

- Often, a sudden change in the entropy indicates a phase change in the underlying system, for example a DDoS (Distributed Denial of Service) attack [5]. To this end, it typically suffices to bound the entropy, since its precise value is not required.
- A good measure of similarity between two datasets is the difference between their aggregated and individual entropies. For example, if two collections of text are of a similar nature, their aggregate entropy will be similar to the individual ones, and if they are different, the aggregated entropy will be substantially larger. Here, too, it suffices to approximate the global entropy, or bound it from above or below in order to reach a decision.

Guided by such challenges, we develop communication-efficient algorithms for bounding and approximating the global entropy, which are organized as follows:

In Section 3, we present algorithms for bounding the global entropy, with low communication. Some results on real and synthetic data are also provided.

In Section 4, a novel algorithm is provided for approximating the global entropy. It is tailored to treat the cases in which the algorithm in Section 3 underperforms.

## 2. Previous Work

The problem of reducing communication overhead in distributed systems is very important both from the practical and theoretical points of view. Applications abound, for example distributed graphs [2,6] and distributed machine learning [3]. Research close to ours in spirit [7] deals with the following scenario: a system is given consisting of

- Distributed computing nodes denoted by $N_{1}\ldots N_{t}$, with $N_{i}$ holding a “local” data vector $X_{i}$. The nodes can communicate, either directly or via a “coordinator” node.
- A scalar-valued function f(X,Y), defined on pairs of real-valued vectors.

Given the above, the challenge is to approximate the values $f(X_{i},X_{j}),i,j=1\ldots t$ with a low communication overhead; that is, the trivial solution of sending all the local vectors to some computing node is forbidden.

A *sketch* for this type of problem is defined as a structure s(), of size smaller than the dimension of $X_{i}$, which has the following property: knowledge of s(X),s(Y) allows one to approximate f(X,Y) with very high accuracy. An important example [7] is f(X,Y)=〈X,Y〉 (〈X,Y〉 stands for the inner product of X,Y).

There are many types of sketches, for example:

- PCA (Principle Component Analysis) sketch: given a large subset $S\subseteq R^{n}$, one wishes to quickly estimate the distance of vectors from an underlying structure which *S* is sampled from (a famous example is images of a certain type [8]). To this end, *S* is represented by a smaller set, consisting of the dominant eigenvectors of *S*’s scatter matrix, and the distance is estimated by the distance of the vector from the subspace spanned by these vectors.
- In the analysis of streaming data, some important sketches were developed, in order to handle large and dynamic streams, by only preserving salient properties (such as the number of distinct items, frequency, and the norm). It is beyond the scope of this paper to describe these sketches, so we refer to the literature [9].

Sketches are specifically tailored for the task at hand. In our case, X,Y are frequency (probability) vectors, and f(X,Y) is the empirical Shannon entropy of $\left(X+Y\right)/2$. Similarly, one may look at functions defined on larger subsets of $\left\{X_{1},\ldots ,X_{t}\right\}$ (Section 3.4). Our task is therefore to define a sketch s(), such that

- s(X) is much smaller than *X*.
- Knowledge of s(X),s(Y) allows one to approximate the empirical Shannon entropy of $\left(X+Y\right)/2$.

We note here that some work addressed entropy approximation in the Streaming Model  [1,10,11]. Here, as in [7], we are mainly interested in the static scenario, in which the *overall* communication overhead is substantially smaller than the overall data volume. The “geometric monitoring” method [10,11], applied to solve the Distributed Monitoring Problem [1], relies on checking local constraints at the nodes; as long as they hold, the value of some global function, defined on the average of the local streams, is guaranteed to lie in some range. Alas, when the local conditions are violated, the nodes undertake a “synchronization stage” [12], which consists of communicating their local vectors in their entirety (which here we avoid). In the future, we plan to extend the techniques developed here to the distributed streaming scenario.

## 3. Dynamic Bounds and Communication Reduction

In this section, we present algorithms for bounding the entropy of a centralized vector—that is, the mean of several local vectors—by broadcasting a controlled amount of inter-communication between machines. The proposed algorithms for both upper and lower bounds accept the same input and therefore can be run concurrently.

### 3.1. Problem, Motivation, and an Example

This work addresses the following problem:

- Given nodes $N_{i}$, each holding a probability vector $v_{i}$ (i.e., all values are positive and sum to 1), approximate the entropy of the average of $v_{i}$, while maintaining low communication overhead.

Let us start with the simplest possible scenario, which we shall analyze in detail, in order to prepare the ground for the general treatment.

**Example 1.**  *There are two nodes, $N_{1},N_{2}$, and the vectors they hold are of length 3. Assume without loss of generality that $N_{1}$ sends some of its data to $N_{2}$, where “data” consists of a set of pairs (coordinate, value), where “coordinate” is the location of a value of $v_{1}$, and “value” is its numerical value; then, $N_{2}$ attempts to derive an upper bound on the entropy of $\frac{v_{1}+v_{2}}{2}$. Note that vectors of length 2 are hardly interesting, since sending a single datum allows one to compute the other (as they sum to 1); hence, $N_{2}$ will be able to* exactly *compute the entropy.*

*Intuition suggests that $N_{1}$ should relay its largest value to $N_{2}$. While (as we will show later), this is true* on the average, *that is not always the case. Assume that the vectors held by the nodes are*

$$v_{1}=\left(\frac{2}{3},\frac{1}{3},0\right),v_{2}=\left(0,\frac{1}{2},\frac{1}{2}\right)$$

*Assume that $N_{1}$ sends its largest value (and its coordinate) to $N_{2}$. Now, $N_{2}$ knows that (a) the first value of the average vector is $\frac{1}{3}$, and (b) the second and third values of $N_{2}$ sum to $\frac{1}{3}$. That leaves open the possibility that these values are $\frac{1}{6}$ each, which would render the average vector equal to*

$$\left(\frac{1}{3},\frac{1}{3},\frac{1}{3}\right),$$

*hence, the upper bound is equal to the maximal entropy possible, 3ln(3). However, if $N_{1}$ sends its second largest value $\left(\frac{1}{3}\right)$ to $N_{2}$, $N_{2}$ can conclude that the second value of the average vector equals $\frac{5}{12}$; hence, the upper bound on the entropy is strictly smaller than 3ln(3).*

We observe here that the key consideration in determining the upper bound is the distribution of the “slack” corresponding to the unknown values at the other node ($N_{1}$ in this example). The overall size of this “slack” is one-half of the unknown values, and it should be distributed amongst the same set of coordinates in $N_{2}$ after they have been divided by 2.

In contrast to the above “adversarial” example, on average, it is optimal to send the largest value (i.e., it allows one to achieve a lower upper bound). To prove this, we have (numerically) computed the integral of the upper bound over all triplets, both after the largest value and a random value were sent; sending the highest value, on the average, yielded an upper bound lower by 0.041 than sending a random value. More general experiments, for both real and synthetic data, are reported in Section 3.5.

We now address the general scenario. Let us start with a few definitions:

**Notation 1.**  *Let $\left\{X_{1},\ldots ,X_{t}\right\}$ be a set of local vectors held in t nodes $\left\{N_{1},\ldots ,N_{t}\right\}$. Then, $\tilde{X}=\frac{1}{t}\sum_{i=1}^{t}X_{i}$ is the aggregate vector, which in our case is the mean over all local vectors.*

**Notation 2.**  *Let x∈[0,1]. We define the Entropy activation function h(x) by:*

$$h\left(x\right)=\left\{\begin{array}{cc} 0 & \;\mathrm{if}x=0\\ -x\ln x & \;\mathrm{otherwise} \end{array}\right.$$

**Definition 1.**  *Let $X\in R^{n}$ s.t $\forall i:0\leq x_{i}\leq 1$. Then, H(X) denotes the Shannon’s Entropy of X [4], given by*

$$H\left(X\right)=\sum_{i=1}^{n}h\left(x_{i}\right)$$

We will henceforth assume all vectors are of length *n* and behave like *X* in Definition 1, even if it is not explicitly noted. We also assume each value of *X* can be represented by at most *b* bits.

**Notation 3.**  *Let $X_{\mathrm{Local}},X_{\mathrm{Other}}$ denote a probability vector held by a local machine and a probability vector held by a remote machine, respectively.*

In this section, we present algorithms for deciding whether the entropy of an average probability vector that sums to 1 is greater or lesser than a user-defined threshold. Formally, we will address two problems:

1. Determining whether the inequality $H\left(\tilde{X}\right)\leq L$ holds for some user-defined constant *L*.
2. Determining whether the inequality $H\left(\tilde{X}\right)\geq U$ holds for some user-defined constant *U*.

We begin with a lemma which provides the foundation for both the Local Upper Bound (Section 3.2) and Local Lower Bound (Section 3.3) in the following subsections.

While noting that the lemma and its corollary hold for any vector $X\in R^{n}$, our vectors are always frequency vectors and hence sum to 1; the Δ below corresponds to the “slack” added after dividing the respective value by 2, as explained in the discussion of Example 1 above; hence, the values still sum to 1.

**Lemma 1**  (Extrema of Entropy)**.** *Let $X=\left(x_{1},\ldots ,x_{n}\right)\in R^{n}$ s.t. $\forall i,x_{i}\geq 0$. Let Δ be a positive number, and let i,j be two distinct coordinates of X.*

- *Let $X^{i}=\left(x_{1},\ldots ,x_{i}+\Delta ,\ldots ,x_{n}\right)$;*
- *Let $X^{j}=\left(x_{1},\ldots ,x_{j}+\Delta ,\ldots ,x_{n}\right)$.*

*If $x_{i}<x_{j}$, then $H\left(X^{i}\right)>H\left(X^{j}\right)$.*

**Proof.**  To establish $H\left(X^{i}\right)-H\left(X^{j}\right)>0$, then since H(X) is coordinate-wise additive, it suffices to show that:

$$h\left(x_{i}+\Delta \right)-h\left(x_{i}\right)>h\left(x_{j}+\Delta \right)-h\left(x_{j}\right)\;.$$

Using the observation that $h^{\prime }\left(x\right)=-\ln x-1$, which is strictly decreasing, we divide the proof into two cases depending on the relation between $x_{i}+\Delta$ and $x_{j}$:

1. $x_{i}+\Delta \leq x_{j}$:
  Since $x_{i}<x_{i}+\Delta \leq x_{j}<x_{j}+\Delta$, the intervals $\left(x_{i},x_{i}+\Delta \right)$ and $\left(x_{j},x_{j}+\Delta \right)$ are disjoint. By applying the Lagrange Mean Value Theorem, for some $c_{1}\in \left(x_{i},x_{i}+\Delta \right)$ and $c_{2}\in \left(x_{j},x_{j}+\Delta \right)$:
  $$\begin{array}{cc} h^{\prime }\left(c_{1}\right) & =\frac{h\left(x_{i}+\Delta \right)-h\left(x_{i}\right)}{\Delta }\\ h^{\prime }\left(c_{2}\right) & =\frac{h\left(x_{j}+\Delta \right)-h\left(x_{j}\right)}{\Delta } \end{array}$$
  Since $h^{\prime }\left(\right)$ is decreasing and $c_{1}<c_{2}$, we immediately obtain that $h^{\prime }\left(c_{1}\right)>h^{\prime }\left(c_{2}\right)$. It follows that:
  $$\begin{array}{cc} \frac{h\left(x_{i}+\Delta \right)-h\left(x_{i}\right)}{\Delta }=h^{\prime }\left(c_{1}\right)\; & >h^{\prime }\left(c_{2}\right)=\frac{h\left(x_{j}+\Delta \right)-h\left(x_{j}\right)}{\Delta }\\ h\left(x_{i}+\Delta \right)-h\left(x_{i}\right)\; & >h\left(x_{j}+\Delta \right)-h\left(x_{j}\right) \end{array}$$
2. $x_{i}+\Delta >x_{j}$:
  Observing the disjoint intervals $\left(x_{i},x_{j}\right)$, $\left(x_{i}+\Delta ,x_{j}+\Delta \right)$. The sought inequality, following the Lagrange Mean Value Theorem for $c_{1}\in \left(x_{i},x_{j}\right),\;c_{2}\in \left(x_{i}+\Delta ,x_{j}+\Delta \right)$, as in the case above, is:
  $$\begin{array}{cc} \frac{h\left(x_{j}\right)-h\left(x_{i}\right)}{\Delta }=h^{\prime }\left(c_{1}\right) & >h^{\prime }\left(c_{2}\right)=\frac{h\left(x_{j}+\Delta \right)-h\left(x_{i}+\Delta \right)}{\Delta }\\ h\left(x_{i}+\Delta \right)-h\left(x_{i}\right) & >h\left(x_{j}+\Delta \right)-h\left(x_{j}\right) \end{array}$$

   □

**Corollary 1.**  *Given a probability vector X, and Δ>0, the following properties hold:*

*1.*  *If Δ is added to any value of X, the maximal increase of its entropy will occur when Δ is added to the minimal value of X.*
*2.*  *If Δ is added to any value of X, the minimal increase of its entropy will occur when Δ is added to the maximal value of X.*

### 3.2. Upper Bound

While ln(n) is a trivial upper bound to the entropy, and does not require any communication to agree upon, we can develop a more efficient alternative while incurring a small communication overhead. Let $X,S_{k}\left(X\right)$ denote a probability vector and a *k*-sized ordered subset of *X*’s *k* largest values, respectively. Hence, let local nodes broadcast the following two ordered sets:

1. $S_{k}\left(X\right)=$ ordered set of largest *k* values of *X*;
2. $C_{k}\left(X\right)=$ the coordinates of the values in $S_{k}\left(X\right)$, or formally $\left\{i|x_{i}\in S_{k}\left(X\right)\right\}$.

Each of these messages costs at most $k(b+\log_{2}n)$ bits: *b* for each value and $\log_{2}n$ for each corresponding coordinate. By sending these subsets of values and coordinates, local machines can immediately obtain the following information regarding the local vector *X* from which $S_{k}\left(X\right),C_{k}\left(X\right)$ were sent:

- The sum of all values not in $S_{k}\left(X\right)$, i.e., $1-\sum_{x\in S_{k}\left(X\right)}x$, will be referred to as the *mass* of the local vector that remains available to be distributed among coordinates. It will be denoted by *m* in the following algorithms.
- $\max\left\{X\backslash S_{k}\left(X\right)\right\}\leq \min\left\{S_{k}\left(X\right)\right\}$, since $S_{k}\left(X\right)$ contains the largest values of *X* (where $X\backslash S_{k}\left(X\right)$ denotes set difference).

We next suggest an algorithm for a local machine with local probability vector $X_{\mathrm{Local}}$ to compute the strict upper bound for $\tilde{X}$, which is the aggregated data of both $X_{\mathrm{Local}}$ and $X_{\mathrm{Other}}$, which is a probability vector that is not accessible to the machine. The remote machine broadcasts $S_{k}\left(X_{\mathrm{Other}}\right)$ and $C_{k}\left(X_{\mathrm{Other}}\right)$ for some predetermined *k*.

The algorithm constructs the unknown subset of the remote machine that ensures the centralized entropy is maximized, or formally ${\mathrm{argmax}}_{X}H\left(X_{\mathrm{Local}}+X\right)$, while maintaining feasible constraints. We view this problem as an instance of constrained optimization, where our target function is the global entropy, and the constraints are given by the broadcast set of $S_{K}\left(X\right)$ and its sum. The main tool is Corollary 1 for every coordinate of $X_{\mathrm{Local}}$.

Before we present the algorithm, we note two extreme cases, which instantly produce an upper bound without the need to algorithmically compute it:

1. $\sum_{x\in S_{k}\left(X\right)}x\approx 1$. In this case, most (or all) the information of *X* is broadcast by the message, and the entropy can be computed accurately without need for a bound.
2. $1-\sum_{x\in S_{k}\left(X\right)}x\geq \sum_{x_{i}\in X_{\mathrm{Local}}}x_{max}-x_{i}$, where $x_{max}=\max\left\{X_{\mathrm{Local}}\right\}$. In this case, there is no need to run the proposed algorithm; the constraint maximization will always result is an “optimal” target—the uniform vector with the value $x_{max}+\frac{1}{n}\left(m-\sum_{x_{i}\in X_{\mathrm{Local}}}x_{max}-x_{i}\right)$, whose entropy we know is maximal w.r.t its sum.

**Theorem 1.**  *Algorithm 1 runs in $O(n^{2})$ time and returns an upper bound on the entropy of $\tilde{X}=\frac{1}{2}\left(X_{\mathrm{Local}}+X_{\mathrm{Other}}\right)$.*

**Algorithm 1:** Upper Entropy Bound for Two Nodes

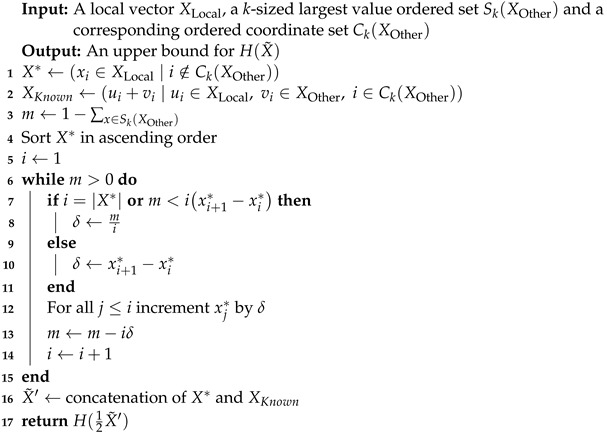

**Proof.**  Let $n^{\prime }$ be the length of $X^{*}$, which equals n−k. In each loop iteration, the algorithm increments no more than $n^{\prime }$ values of $X^{*}$, and since there are $n^{\prime }$ coordinates of $X^{*}$, it will perform at most $n^{\prime }$ steps. Hence, the bound of $O(n^{2})$ runtime follows.

Let $X^{*}=\left(x_{1},\ldots ,x_{n^{\prime }}\right)$ be the initial vector as noted in line 3, and let *Y* denote the same by the end of the while loop, i.e., after the condition m=0 is met. Let the coordinates of *Y* be arranged in ascending order, which has no effect on its entropy: $Y=\left(y_{1},y_{2},\ldots ,y_{t},y_{t+1},\ldots ,y_{n^{\prime }}\right)$. Since at every loop iteration, all minimal coordinates are incremented simultaneously, there exists some coordinate *t* such that for all i<t, $y_{i}$ equals *c*, and for all i>t, $y_{i}$ is strictly greater than *c*. Hence, we can view *Y* as a concatenation of the two vectors $\left(Y_{L},Y_{R}\right)$ as defined below:

- $Y_{L}=\left(y_{1},\ldots ,y_{t}\right)=\left(c,\ldots ,c\right)$;
- $Y_{R}=\left(y_{t+1},\ldots ,y_{n^{\prime }}\right)$.

Let $s\left(X\right)$ denote the sum of *X*. It now suffices to show that any vector *Z* that sums to $s\left(X^{*}\right)+m$ and can be achieved by performing only additions to $X^{*}$ has a lesser or equal entropy value than *Y*. Let *Z* denote such a vector for every value of which $z_{i}$ satisfies $z_{i}\geq x_{i}$. Let $Z=(Z_{L},Z_{R})$, where $Z_{L}=\left(z_{1},\ldots ,z_{t}\right),Z_{R}=\left(z_{t+1},\ldots ,z_{n^{\prime }}\right)$ for the same *t* as defined above.

Since it holds that $s\left(Z\right)=s\left(Z_{L}\right)+s\left(Z_{R}\right)=s\left(Y_{L}\right)+s\left(Y_{R}\right)=s\left(Y\right)$, we examine the following cases:

- $s\left(Z_{L}\right)=s\left(Y_{L}\right),\;s\left(Z_{R}\right)=s\left(Y_{R}\right)$: Note that $Z_{R}=Y_{R}$, since their sum is equal, and $Y_{R}$ has had no further additions. In addition, since $s\left(Z_{L}\right)=s\left(Y_{L}\right)=c\cdot Y_{L}$ and $Y_{L}$ is the uniform vector, $H\left(Z_{L}\right)\leq H\left(Y_{L}\right)$. It follows that $H\left(Z_{L}\right)+H\left(Z_{R}\right)\leq$$H\left(Y_{L}\right)+H\left(Y_{R}\right)$.
- $s\left(Z_{L}\right)<s\left(Y_{L}\right),\;s\left(Z_{R}\right)>s\left(Y_{R}\right)$: there exists a subset $\left(z_{i_{1}},\ldots ,z_{i_{\ell }}\right)$$\subseteq Z_{R}$ for which every $z_{i_{j}}$ is greater than the corresponding value $y_{i_{j}}$ of $Y_{R}$. Let $\delta_{i_{j}}=z_{i_{j}}-y_{i_{j}}$. For every $\delta_{i_{j}}$, there exists a value $z_{\ell }$ in $Z_{L}$ s.t $z_{\ell }<y_{i_{j}}<z_{i_{j}}$, since $s\left(Z_{L}\right)<s\left(Y_{L}\right)=c\cdot Y_{L}<y_{i_{j}}\cdot Y_{L}$. Let $Z^{\prime }$ be *Z* after $\delta_{i_{j}}$ is subtracted from each $z_{i_{j}}$ and added to some $z_{\ell }\in Z_{L}$ as described above. By Lemma 1, $H\left(Z^{\prime }\right)>H\left(Z\right)$. It also holds that $Z_{R}^{\prime }=Y_{R}$, and that $H\left(Z_{L}^{\prime }\right)\leq H\left(Y_{L}\right)$, since they both sum to $c\cdot Y_{L}$, and $Y_{L}$ is a uniform vector (whose entropy is maximal). Therefore, $H\left(Z\right)<H\left(Z^{\prime }\right)\leq H\left(Y\right)$.
- $s\left(Z_{L}\right)>s\left(Y_{L}\right),\;s\left(Z_{R}\right)<s\left(Y_{R}\right)$: this case can be immediately omitted; it is an impossibility to feasibly subtract a value from $Z_{R}$ or increase the value of $s\left(Z_{L}\right)$ above $s\left(Y_{L}\right)$.

Therefore, we have proven for any vector *Z*, it holds that H(Z)≤H(Y).    □

Next, we suggest a more time-efficient algorithm than Algorithm 1 that achieves an equivalent bound, with a runtime of $O\left(n\log n\right)$. Suppose *c* is the maximal threshold all values of the local vectors can be incremented to without exceeding the sum of the values from the remote vector, *m*. Then, if we define the sorted coordinates of $X^{*}$ to be $x_{1},\ldots ,x_{n^{\prime }}$, there is some coordinate *t* such that $x_{t}\leq c\leq x_{t+1}$.

By performing a binary search on the the coordinate *t* of the local vector as described above, we can efficiently find that $x_{t}$ as described in the algorithm below.

**Theorem 2.**  *Algorithm 2 runs in O(nlogn) time and returns an upper bound for the entropy of $\tilde{X}=\frac{1}{2}\left(X_{\mathrm{Local}}+X_{\mathrm{Other}}\right)$.*

**Algorithm 2:** Binary Search Upper Entropy Bound for Two Nodes

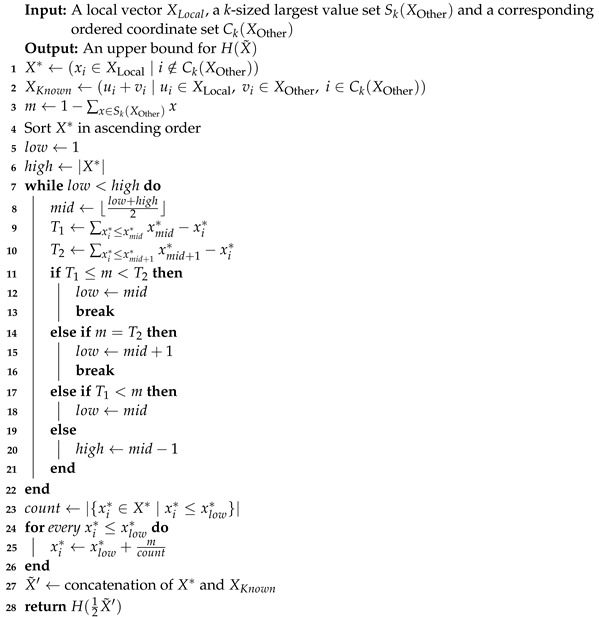

**Proof.**  The algorithm begins by sorting $X^{*}$, which costs $O\left(n\log n\right)$ and follows by performing a binary search on a range of size $n^{\prime }$, wherein a single step requires O(n) operations. Therefore, its runtime is $O\left(n\log n\right)$.

Since the vector $X^{*}$ constructed by this algorithm is equivalent to the vector which Algorithm 1 computes, the proof of correctness is the same as the proof of Theorem 1.    □

It will be noted that the upper bound given by Algorithms 1 and 2 can be further improved by using the feasibility constraint upon $X^{*}$. It is possible to increase a coordinate of $X^{*}$ by a value larger than $\min\{S_{K}\left(X_{\mathrm{Other}}\right)\}$, particularly if for some coordinate *i*, the inequality $x_{i+1}^{*}-x_{i}^{*}>\min\left\{S_{K}\left(X_{\mathrm{Other}}\right)\right\}$ holds. In order to keep the core algorithms simple, we will address this formally in Appendix A by proposing an improvement to the algorithms above, such that the bound will indeed by tight.

### 3.3. Lower Bound

We now turn to discuss a communication-efficient solution for computing a tight lower bound for the entropy of a global vector. As with the upper bound, this problem is an instance of constrained optimization, only that here, our target is to find the minimum. As with the Upper Bound (Section 3.2), we use the same message containing $C_{k}\left(X\right)$ and $S_{k}\left(X\right)$, for a remote vector *X*.

**Property 1.**  Let $n^{\prime }$ denote $X^{*}$, sup denote $\min\{S_{k}\left(X_{\mathrm{Other}}\right)\}$ and m denote $1-\sum_{x\in S_{k}\left(X_{\mathrm{Other}}\right)}x$, as used in Algorithm 3. Then, $n^{\prime }\cdot sup\geq m$.

**Algorithm 3:** Lower Entropy Bound for Two Nodes

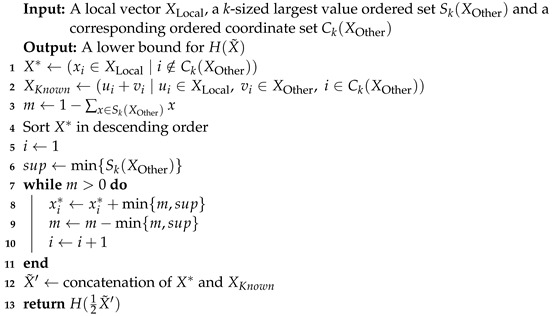

**Proof.**  Using the definitions, we obtain the following inequality:

$$X^{*}\cdot \min\left\{S_{k}\left(X_{\mathrm{Other}}\right)\right\}\geq 1-\sum_{x\in S_{k}\left(X_{\mathrm{Other}}\right)}x\;$$

which holds, since:

$$\sum_{x\in S_{k}\left(X_{\mathrm{Other}}\right)}x+X^{*}\cdot \min\left\{S_{k}\left(X_{\mathrm{Other}}\right)\right\}\geq \sum_{x\in X_{\mathrm{Other}}}x=1\;.$$

   □

**Theorem 3.**  *Algorithm 3 runs in O(nlogn) time and returns a tight, lower bound for the entropy of $\tilde{X}=\frac{1}{2}\left(X_{\mathrm{Local}}+X_{\mathrm{Other}}\right)$*

**Proof.**  After sorting the vector, we iteratively increment no more than $n^{\prime }=X^{*}\leq n$ coordinates since $n^{\prime }\cdot sup\geq m$ by Property 1; hence, the total runtime is $O\left(n\log n\right)$.

To prove the correctness of the bound, it suffices to examine our loop step; it is clear we must add a total sum of *m* to any of $X^{*}$’s coordinates, and we *cannot* increment a single coordinate by more than sup—since we know all remaining values of the unknown vector *X* are lesser or equal to sup.

The algorithm increments the maximal values of $X^{*}$ by sup, which by Corollary 1 incurs the minimal entropy gain to $X^{*}$. Due to the fact that the entropy is coordinate-wise additive, the ”greedy” approach which minimizes over coordinates separately reaches the global minimum.    □

**Figure 1:**
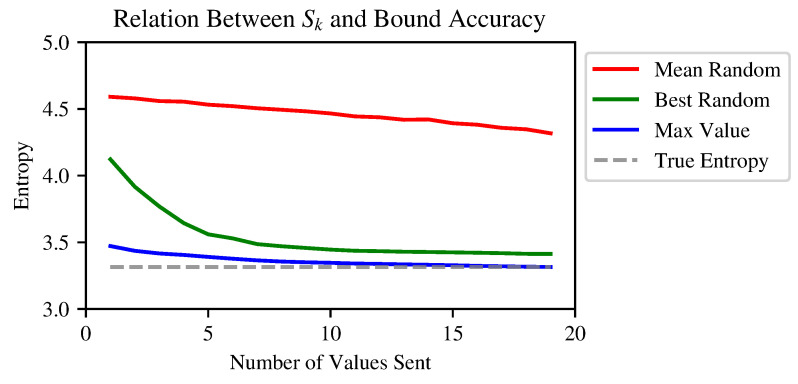
Comparison of three upper bounds as a function of the number of values sent. The red plot corresponds to an average of random selections of values from the remote vector; the green plot represents the best results (i.e., lower upper bounds) from 10,000 random selections; the blue plot represents the algorithm described here, where $S_{k}\left(X\right)$ consists of the largest values. The vectors have a length of 100, and their values are sampled from a half-normal distribution with standard deviation 0.02 and then normalized to sum 1.

In Figure 1, the proposed algorithm, and the bounds computed using the algorithms described herein, are compared to the bounds derived after sending a random subset of coordinate–value pairs, as well as sending many random subsets and choosing the minimal resulting bound. In Section 3.5, more extensive experiments are reported.

### 3.4. Multiparty Bounds

When considering the scale and variability of modern distributed systems, an algorithm that supports multiple machines and incurs a low communication overhead is desirable.

We next suggest a few modifications in order to generalize Algorithms 1–3, for the upper and lower bounds of entropy centralized across t+1 nodes. We denote $X_{i}$ as the vector of machine *i*, and in a manner similar to Section 3.2, $S_{k}\left(X_{i}\right),C_{k}\left(X_{i}\right)$ are the ordered sets of the *k* maximum values and their coordinates, respectively. Typically, the coordinates of $C_{k}\left(X_{i}\right)$ and $C_{k}\left(X_{j}\right)$ will be disjoint, in which case each machine will have to broadcast its missing coordinates. The additional communication may cost us up to $tk(b+\log_{2}n)$ bits. We hereby assume a second round of communication occurs, and that $\left(S_{k}\left(X_{1}\right),\ldots ,S_{K}\left(X_{t}\right)\right)$, as well as $\left(C_{k}\left(X_{1}\right),\ldots ,C_{K}\left(X_{t}\right)\right)$ include the same coordinates.

Below, we list the modifications to be made to the previous algorithms for the multiparty case. These changes are similar for all three algorithms.

- Input: In addition to the local vector $X_{Local}$, the *k*-sized largest value sets $S_{k}\left(X_{1}\right),\ldots S_{k}\left(X_{t}\right)$ and corresponding ordered coordinate sets $C_{k}\left(X_{1}\right),\ldots ,C_{k}\left(X_{t}\right)$—instead of single sets.
- The sum to be added to all coordinates of $X^{*}$, *m* will be $t-\sum_{i=1}^{t}\left(\sum_{x\in S_{k}\left(X_{i}\right)}x\right)$, since there are *t* local vectors to process and construct, while local vector $X_{i}$ contributes $1-\sum_{x\in X_{i}}x$.
- The return value is now $H(\frac{1}{t+1}{\tilde{X}}^{\prime })$, since we have summed *t* additional vectors into $X^{*}$ and $X_{Known}$.

### 3.5. Experimental Results

To evaluate our algorithms, we tested them on both real and synthetic probability vectors. We now describe the methods and data used to perform our experiments and analyze the results.

**Figure 2:**
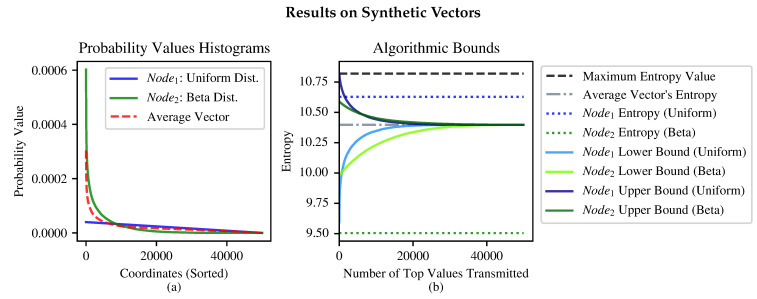
Algorithmic bounds for the empirical entropy on synthetic probability vector of dimension 50 k. (**a**) depicts the distributions of the generated vectors: a normalized uniform distribution (i.e., each value is randomly selected from U[0,1], and then their sum is normalized to 1) which for brevity we refer to as “uniform” at $Node_{1}$, and a beta distribution at $Node_{2}$ with parameters α=0.2,β=100. The dashed line is the average vector of the two. (**b**) demonstrates the locally calculated upper bound and lower bound for different numbers of top values transmitted (*k*) in Algorithms 2 and 3.

**Figure 3:**
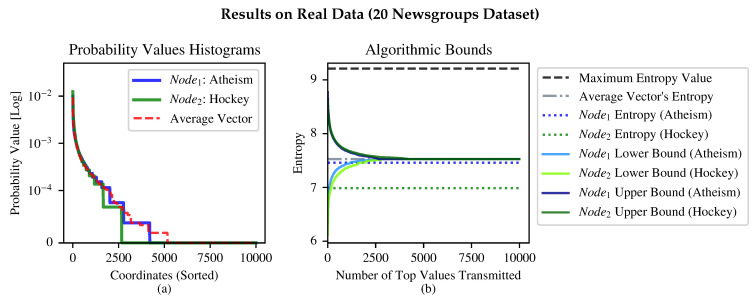
Algorithmic bounds between token frequency vectors of atheism-themed newsgroups and hockey-themed newsgroups. (**a**) depicts the histogram of the tokens of the accumulation of the first 200 articles in each theme. Note that the histogram’s coordinates are organized in descending order of each vector’s values *separately*; thus, the average vector may be larger, for some values, from both $Node_{1}$ and $Node_{2}$. (**b**) demonstrates the locally computed upper bound and lower bound for different numbers of top values transmitted (*k*) in Algorithms 2 and 3.

In Figure 2 and Figure 3, we simulated the upper bound algorithm (Algorithm 2) and the lower bound algorithm (Algorithm 3). Figure 2b depicts a simulation of the algorithms on two randomly generated vectors: $Node_{1}$ with uniform distribution and $Node_{2}$ with beta distribution. The probability vectors of $Node_{1}$ and $Node_{2}$ are shown in Figure 2a. Note that as depicted in Figure 2b, the algorithms’ results in each node are determined by the distribution of the local probability vectors. That is because the more probability *mass* is transmitted, the tighter the bounds become, and the quicker it converges with respect to *k*. As illustrated, in the bounds of $Node_{1}$, which receives $Node_{2}$’s maximal beta distribution’s probability values, the bounds converge quickly with respect to *k*. In contrast, the bounds that are computed at $Node_{2}$, which receives the maximal values of the uniform distribution of $Node_{1}$, converge slowly to the real entropy. This is due to the fact $Node_{2}$ does not gain much information from $Node_{1}$.

Fortunately, the difference between the bounds of $Node_{1}$ and $Node_{2}$ is an advantage to our proposed algorithms; we can compare them and use the better one simply by comparing the bounds (which requires transmitting only one scalar).

Another interesting observation can be drawn from Figure 2b; $Node_{1}$’s lower bound is already quite close to the global entropy for very small *k* values. The algorithm works well here since the maximum value of $Node_{2}$ is not large, which in turn enables the algorithm to reach a tighter bound.

Figure 3b illustrates our experiments on the 20 Newsgroups Dataset [13] which includes about 20,000 newsgroup documents for 20 different topics. We measured the entropy of token frequency vectors (A vector where each value corresponds to the frequency of a word or token in the document).  from the atheism-themed newsgroups and the hockey-themed newsgroups. To do so, we took the top 10,000 occurring tokens and created token frequency vectors on the first 200 articles from the atheism theme and the hockey theme. The visual illustration of the (sorted) tokens frequency is in Figure 3a. As can be observed, the atheism newsgroups is more verbally rich than the hockey newsgroup, having more words which are unique to it.

As demonstrated in Figure 3b, the upper bound computed by both nodes is almost the same. However, for the lower bound, $Node_{1}$ (atheism) converges faster to the real entropy as we increase the parameter *k* of the algorithm. We attribute that to the denser token histogram of the hockey theme; thus, more “probability mass” is transmitted for the same *k*.

**Figure 4:**
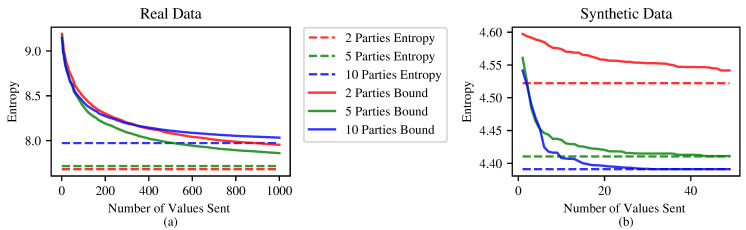
An example of the multiparty upper bound on real and synthetic data. (**a**) For the real data, we used vectors from the newsgroups dataset; each vector is of length 10,000. (**b**) The synthetic data are sampled from half-normal distribution; each vector is of length 1000.

Figure 4 presents results for the multiparty case, as discussed in Section 3.4.

**Figure 5:**
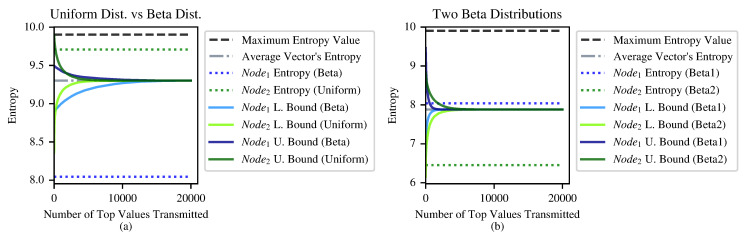
Rate of convergence of the dynamic bound algorithms in Section 3 to the real entropy values as a function of communication overhead. (**a**) $Node_{2}$ obeys a uniform distribution, and $Node_{1}$ obeys a beta distribution with α=0.1, β=100. (**b**) Both nodes obey a beta distribution, one with α=0.1, β=100 and the other with α=0.02, β=100.

To conclude, the distribution of the probability vectors directly affects the tightness of the bounds. The less concentrated the probability vectors are, the less information we can send for every *k*; hence, the bounds become less tight, as demonstrated in Figure 5. A solution for this case is presented in Section 4.

## 4. Entropy Approximation

The algorithms described in Section 3 perform better in terms of communication overhead when there are a few relatively large values in the local frequency vectors, i.e., a substantial percentage of the overall “probability mass” resides in a relatively small percentage of the vectors’ values. However, in the case in which the vectors are “flat”—that is, their distribution approaches a uniform one—the nodes will have to exchange many values in order to reach tight bounds on the overall entropy; see Figure 5. In this section, we offer a probabilistic solution to this problem.

Assume that two nodes $N_{1},N_{2}$ hold vectors X,Y, and the goal is to approximate the entropy of the average vector $\frac{X+Y}{2}$, with a small communication overhead, relative to *n*, the length of the vectors.

One solution, which was applied in previous work on monitoring entropy [10], is to use *sketches*. This popular technique found many applications in computer science, for example, for computations over distributed data [7]. A well-known sketch for entropy, which we describe in Section 4.1, is presented in [14]; see also [15].

Here we use a different sketch, which, for our purposes, performed better than the sketch presented in [14]. In resemblance to Section 3, the two nodes first exchange all values which are greater or equal to a threshold ε, whose value is determined by a communication/accuracy trade-off. Hence, we assume hereafter that all values are smaller than ε. Next, choose a polynomial approximation, of degree at least 2, over the interval [0,ε], to the function h(t)≜−tln(t). Assuming in the meanwhile a degree 2 approximation, denote it by $At^{2}+Bt+C$. The proposed method is oblivious to the choice of this approximation; we have used the approach of minimizing

$$\int_{0}^{\varepsilon }{\left(f\left(t\right)-\left(At^{2}+Bt+C\right)\right)}^{2}dt\;,$$

which allows a closed-form solution

$$A=-\frac{5}{4\varepsilon },\;B=-\ln\left(\varepsilon \right)+\frac{13}{12},\;C=\frac{\varepsilon }{8}\;.$$

Using this quadratic function, we can approximate the entropy of the average vector $\frac{X+Y}{2}$ by

$$\begin{array}{cc} \; & \sum_{i=1}^{n}\left(A{\left(\frac{X_{i}+Y_{i}}{2}\right)}^{2}+B\left(\frac{X_{i}+Y_{i}}{2}\right)+C\right)=\\ \frac{A}{4} & \sum_{i=1}^{n}\left(X_{i}^{2}+Y_{i}^{2}\right)+\frac{A}{2}\sum_{i=1}^{n}X_{i}Y_{i}+\frac{B}{2}\sum_{i=1}^{n}\left(X_{i}+Y_{i}\right)+nC\;. \end{array}$$

Note that with the exception of the term $\sum_{i=1}^{n}X_{i}Y_{i}$, all terms can be computed locally and require O(1) communication overhead to transmit. Thus, it only remains to approximate the term $\sum_{i}X_{i}Y_{i}$, which equals the inner product 〈X,Y〉. To this end, we can apply an approximation based on the famed *Johnson–Lindenstrauss Lemma* [16], which is defined as follows:

$$\langle X,Y\rangle \approx \frac{1}{d}\sum_{i=1}^{d}\langle X,R_{i}\rangle \langle Y,R_{i}\rangle \;;$$

where $R_{i}$ are independent random vectors with all values i.i.d standard normal variables, which are generated by a pre-agreed upon random seed, and thus require no communication. A direct calculation yields that this estimate has expectation 〈X,Y〉 (i.e., is unbiased) and its variance equals

$$\frac{{∥X∥}^{2}{∥Y∥}^{2}+\langle X,Y\rangle }{d}\;.$$

Similarly, we can apply higher-order approximations. For a cubic approximation, we obtain a more complicated but identical in spirit sketch, which requires an approximation of the expressions $\sum_{i=1}^{n}X_{i}^{2}Y_{i},\sum_{i=1}^{n}X_{i}Y_{i}^{2}$; this, too, can be achieved by applying the estimate above, since these quantities can also be represented as inner products of “local vectors”: for example, $\sum_{i=1}^{n}X_{i}^{2}Y_{i}=\langle X^{2},Y\rangle$, where ${\left(X^{2}\right)}_{i}≜X_{i}^{2}$.

**Figure 6:**
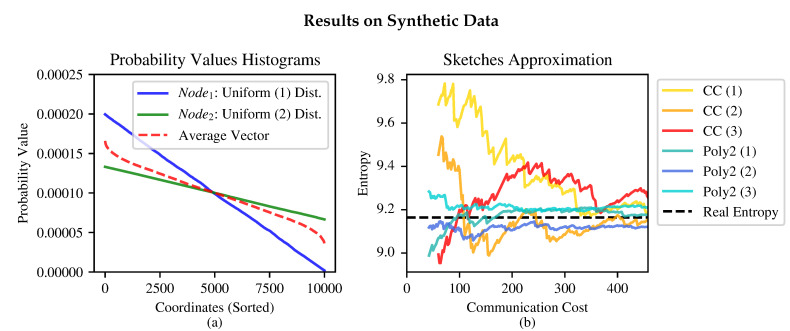
Comparison of the Poly2 and CC sketches for approximating the empirical Shannon entropy. (**a**) illustrates the synthetic probability vectors that were generated to perform the comparison. (**b**) compares the Poly2 sketch to the CC sketch for varying sketch sizes. The comparison was made using three different random seeds for the sketches. We used the value ε=0.0002.

**Figure 7:**
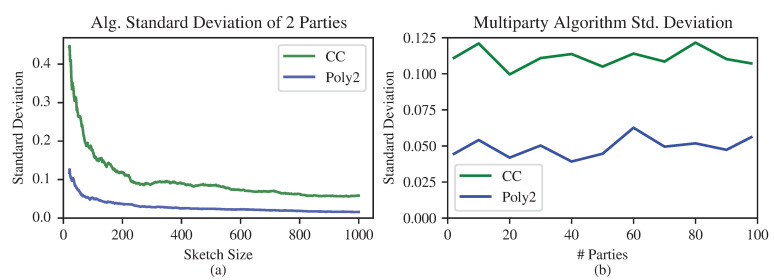
(**a**): standard deviation of the error of the CC and Poly2 sketches, for two parties and varying sketch size. The experiments were performed on a vector of dimension 10000 with uniform distribution, which was followed by normalization to sum 1. The standard deviation was calculated on 50 sketches for each sketch size. (**b**): comparison of the standard deviation of CC and Poly2 sketches in the multiparty scenario for fixed sketch size and varying number of parties. The experiments were performed for an i.i.d random vector distribution of dimension 5000 with sketch size 200 and ε=0.0002.

Some results for two nodes are presented in Figure 6, in which the proposed sketch is compared to the one in [14] (see Section 4.1). Extending the sketch to the multiparty scenario is straightforward; results are presented in Figure 7b.

### 4.1. The Clifford-Cosma Sketch

We compare our sketch to an entropy sketch proposed in  [14]. The sketch is a linear projection of the probability vector. The linear projection is performed by a multiplication matrix with i.i.d elements drawn from F(x;1,−1,π/2,0). The entropy approximation of the *d*-dimensional linear projected vector $\left(y_{1},\ldots ,y_{d}\right)$ is:

$$\tilde{H}\left(y_{1},\ldots ,y_{d}\right)=\ln\left(d\right)-\ln\left(\sum_{i=1}^{d}e^{y_{i}}\right)\;.$$

### 4.2. Sketch Evaluation

We now compare the proposed sketch to the one in [14], which is denoted “CC”. The proposed quadratic sketch is denoted “Poly2”.

## 5. Conclusions

We have presented novel communication-efficient algorithms for bounding and approximating the entropy in a distributed setting. The algorithms were tested on real and synthetic data, yielding a substantial reduction in communication overhead. Future work will address both sketch-based techniques and further development of the dynamic bound algorithms presented here. In addition, we intend to address the efficient distributed computation of other functions.

## Data Availability

The 20 Newsgroups Dataset, which can be found in this link.

## Appendix A. Improving the Upper Bound

Let us recall Algorithms 1 and 2 from Section 3.2. As mentioned there, the computed bound can be improved; we now present this improvement as a generalized algorithm that achieves a tight upper bound. The algorithm below can be run following the upper bound algorithms (either of them), after $X^{*}$ is computed, or as an external procedure that accepts $X^{*}$, *m* and sup as input. We show the former option:

**Algorithm A1:** Tight Entropy Upper Bound for Two Nodes
